# Dietary Fiber Supplementation in Replacement Gilts Improves the Reproductive Performance From the Second to Fifth Parities

**DOI:** 10.3389/fvets.2022.839926

**Published:** 2022-04-26

**Authors:** Yong Zhuo, Lun Hua, Lianqiang Che, Zhengfeng Fang, Yan Lin, Shengyu Xu, Jianping Wang, Jian Li, Bin Feng, De Wu

**Affiliations:** Institute of Animal Nutrition and Key Laboratory for Animal Disease-Resistance Nutrition of the Ministry of Education of China, Sichuan Agricultural University, Chengdu, China

**Keywords:** anti-mullerian hormone, gilts, soluble fibers, sows, lifetime performance

## Abstract

This study examined the effects of soluble fiber (SF) supplementation (0.8%), containing 17.4% rhamnose, 4.1% fucose, 11.1% arabinose, 30.6% xylose, and 16.4% galactose during the prepubescent phase on the subsequent performance from the second to fifth parities. After the first parity, 56 and 55 post-weaning sows in the control (CON) and SF groups had their reproductive performance monitored in succeeding parities. Circulating concentrations of anti-mullerian hormone (AMH) were greater in the SF group than in the CON group at 205 d of age and the first post-weaning day (*p* < 0.05). The SF treatment at the prepubescent phase resulted in an enhanced reproductive performance from parities three to five. In Parity three, the SF sows had 1.32 total born (*p* = 0.044), 1.43 born alive (*p* = 0.023) and 1.40 born effective, which was significantly more than in the CON group (*p* = 0.022). In Parity four, the SF sows had 1.1 total born (*p* = 0.058), 1.28 born alive (*p* = 0.019), and 1.06 born effective, significantly more than in the CON group (*p* = 0.049). In Parity five, the SF gilts had 1.43 total born (*p* = 0.075), 1.53 born alive (*p* = 0.067) and 1.65 born effective, significantly more than in the CON group (*p* = 0.020). No effects were observed for the removal of sows and backfat thickness at the mating in each parity between groups (*p* > 0.05). Collectively, gilts that received an extra 0.8% SF during the prepubescent phase increased their subsequent litter size as breeding sows. These results showed that nutritional decisions at the replacement phase could influence lifetime fertility.

## Introduction

Approximately 50% of the sows on modern swine farms in Europe (1), USA (2), and China (3) are culled each year, and these breeding herds have to be replenished with young replacement gilts. The development quality of replacement gilts, therefore, plays a key role in affecting the reproductive performance of a sow herd as there is a proven relationship between the age, backfat, and body weight at puberty of gilts and their lifetime fertility. The gilts with an average daily gain of 601–650 g per day during their replacement phase had a greater number of piglets in the second parity than those with an average daily gain of 551–600 g per day (4). Litter size as sows was greater if first puberty was observed between 181 and 200 days as replacement gilts than those with first pubertal age between 150 and 180 days, and between 201 and 220 days (5). It has been shown that reproductive traits such as litter size at the first parity could be used to predict the lifetime productive performance of sows on both southern European Union and Asian commercial farms (6, 7), thus, management factors during the growing phase of gilts were able to affect their subsequent performance both in the short and long terms (8).

In our previous study, dietary supplementation with soluble fiber (SF) reduced the age at puberty in gilts and increased their subsequent reproductive performance as sows at their first parity (9), but it remained unclear whether any beneficial effects could also be shown in the subsequent parities. There is growing evidence stressing the importance of ovarian follicle reservation and quality for a longer reproductive span, which is very important for swine production due to the large culling rate of young sows (10, 11). Researchers in this laboratory revealed that dietary macronutrient balance influenced lifetime fertility by regulating ovarian reservation (12). Anti-Mullerian hormone (AMH), a hormone secreted from the ovarian granulosa cells in the growing follicles, is a predictor of an ovarian follicle pool in different mammal species (13–15), and its concentration in prepubescent gilts could be considered a marker of future reproductive success (16). The inclusion of dietary fiber in the diet of replacement gilts affected the ovarian follicle atresia and the size of a follicle pool (17, 18), forming a basis for the control of long-term fertility as breeding sows. However, it remains unclear whether dietary fiber supplementation for replacement gilts could have a long-term positive effect on the reproductive performance as sows, so, by tracing the subsequent performance through parities two to five, the objective of this study was to investigate the effect of dietary SF supplementation during the replacement phase on their subsequent reproductive performances.

## Methods

All experimental procedures were approved by the Animal Care and Use Committee of Sichuan Agricultural University and were in accordance with the National Research Council's Guide for the Care and Use of Laboratory Animals.

### Animals and Experimental Design

The current experiment was a follow-up study of previous research (9). A total of 136 Landrace × Yorkshire gilts with similar mean body weights (BW) of 60.59 ± 7.02 kg and a mean age of 140 ± 10 days were randomly divided into two groups as a control (CON) group to be fed a CON diet or an SF group fed a CON diet supplemented with 0.8% SF at the expense of corn. The CON diet was formulated based on corn and soybean meals to contain 3.37 Mcal of DE/kg, 17.30% crude protein, and 0.92% lysine. The SF was obtained from Linseed Biological Tech. Co., Ltd (Xinjiang, China), and contained 17.4% rhamnose, 4.1% fucose, 11.1% arabinose, 30.6% xylose, 16.4% galactose, 3.4% glucose, 8% water, 4% ash, and 3.4% crude protein.

### Gilt Management

All gilts were group reared in eight pens with 17 gilts in each 6 × 7 m pen from the start of the experiment to the age of 205 days with four pens for each diet, followed by 2 × 0.8 m individual housing from the age of 205 days. The mean feed intake was 2.25 ± 0.22 kg/d and 2.21 ± 0.19 kg/d from the start of the experiment to the age of 205 days for CON and SF, respectively. Feed intake from the age of 205 days to mating at the onset of the third estrus was controlled at 2.50 kg/d for both groups. After mating, the sows were fed the same gestation diet, and, after parturition, all the sows were fed the same lactation diet. Finally, 56 and 55 sows were delivered and weaned randomly into the CON and SF groups, respectively, and the subsequent performance of those trial sows was monitored from Parities two to five.

### Post-Weaning Sow Management

The post-weaning sows were fed 3.5 kg/d of lactation diet (Table 1) until the first estrus. The occurrence of estrus of those 111 post-weaning sows was checked daily as previously (9), and they were artificially inseminated two times with fresh, pooled semen from the same boars at 12 and 24 h after the onset of the post-weaning estrus. Immediately after insemination, all the sows were fed the same two-phase gestation diet (Table 1), where gestation diet I was provided at 2.20 kg/d from days 35–90 of gestation, and gestation diet II was provided at 2.6 kg/d from day 91 of gestation to parturition. However, some sows with lower backfat thickness at mating were fed with 10% more feed than the average. The sows were housed in individual 2.20 × 0.65 m gestation stalls from day 1–106 of gestation. On day 107 of gestation, the sows were moved to individual farrowing pens. The sows were fed two times daily at 08:00 and 16:00.

**Table 1:** Composition of diets (% as fed).

**Ingredients**	**CON^**a**^**	**Gestation I**	**Gestation II**
Corn	62.67	64.85	72.95
Soybean meal (43% CP)	22.0	13.5	18.5
Wheat bran	6.0	18.0	5.0
Fish meal (62.5% CP)	3.1	0	0
Soybean oil	3.0	0	0
Fine limestone	1.48	1.15	1.08
CaHPO_4_∙2H_2_O	0.97	1.65	1.72
Feed-grade NaCl	0.4	0.4	0.4
Trace minerals^b^	0.15	0.15	0.15
Vitamins^c^	0.02	0.02	0.02
Choline chloride (50%)	0.15	0.15	0.15
L-Lysine HCl (98.5%)	0.05	0.05	0
DL- Methionine (99%)	0	0.02	0.02
L-Threonine (98.5%)	0	0.05	0
Phytase	0.01	0.01	0.01
Total	100	100	100
**Calculated nutrient content**			
Crude protein (%)	17.3	13.5	14.3
Digestible energy (Mcal/kg)	3.37	3.05	3.2
Total Lysine (%)	0.92	0.65	0.70
Soluble fiber (%)	1.18	1.32	1.29
Insoluble fiber (%)	10.8	12.1	11.6
Calcium (%)	0.9	0.9	0.9
Total phosphorus (%)	0.7	0.72	0.67
Available phosphorus (%)	0.41	0.45	0.45

a *A soluble fiber mixture (0.8%), containing 17.4% rhamnose, 4.1% fucose, 11.1% arabinose, 30.6% xylose, 16.4% galactose, 3.4% glucose, 8% water, 4% ash, and 3.4% crude protein, was added to CON diet at the expense of corn for gilts-fed SF diet. Gestation diet I was provided at 2.20 kg/d from Days 35 to 90 of gestation, and gestation diet II was provided at 2.6 kg/d from Day 91 of gestation to parturition*.

b *Provided per kg of diet: copper, 10 mg, 10 mg, and 20 mg for CON, gestation, and lactation diet, respectively; iron, 80 mg; zinc, 100 mg; manganese, 25 mg; selenium,0.15 mg; iodine,0.14 mg*.

c *Vitamin premix provided per kg of diet: vitamin A, 4,000 IU; vitamin D_3_, 800 IU; vitamin E, 441 IU; menadione,0.5 mg; thiamine, 1. mg; riboflavin, 3.75 mg; vitamin B_6_, 1. mg; vitamin B_12_, 15 μg niacin, 10 mg; D-pantothenic acid, 12 mg; folic acid, 1.3 mg; D-biotin, 200 μg*.

### Post-Delivery Management

After delivery, all the sows were fed the same lactation diets, and the sows were weaned at 21 d of lactation. To avoid the difference of litter size on the body reservation that might cause influences on the subsequent reproduction, the newborn piglets were cross-fostered to have a similar number of suckling of 10 to 11 piglets. A water curtain cooling system was used to control the room temperature at 25 to 27°C and relative humidity at 60 to 70% in summer and a heat booster was used to maintain the room temperature at 16 to 18°C and relative humidity at 45 to 55% in winter. In other months, the room temperature was controlled from 18 to 22°C and relative humidity at 50 to 60%. Throughout the experiment, water was provided *ad libitum*, and artificial light was provided from 07:00 until 19:00 daily.

### Blood Sampling and Hormone Assays

Blood samples were collected randomly from 12 non-pubertal gilts at an average age of 205 d. Piglets were weaned at day 21 of lactation, and the blood samples were collected from 12 sows on day 22 of partition after weaning the 1st parity before the morning feeding. Plasma samples were collected by centrifuging the blood samples at 2,400 *g* for 30 min at 4°C and were then stored at −20°C for future analysis. The plasma AMH levels were detected using the enzyme-linked immunosorbent assay with the commercial kit (CUSABIO Biotech, Wuhan, China) at a 1:2 dilution and following the manufacturer's instructions. The detection range was 1.25–50. ng/ml and the detection sensitivity was 1.25 ng/ml.

### Removal of Sows

In the present study, sows were removed from examination of their reproductive performance as previously described (9, 11). Post-weaning sows with anestrus, failure to become pregnant after being inseminated two times at 12 and 24 h after standing heat or return to estrus, abortions, and mummification were defined as reproductive disorders. The sows that had a small litter born <6 or low-rearing ability were considered as low performance. The sows with illness, such as lameness, undesirable vulval discharge, udder problems, and respiratory disease, as well as other unplanned reasons, were also excluded from the investigation.

### Backfat Thickness and Reproductive Data

The backfat thickness was detected at the P_2_ point six cm off the midline of the last rib using a Lean Meater (Renco-Lean Meater, Minneapolis, MN, USA) at the mating at each parity. At each parity, litter performance, including total piglets born and piglets born alive, was recorded, and the number of stillborn or mummified pigs was also recorded. The piglets with a birth weight 69% lower than the average were classified as intrauterine growth retarded (IUGR) piglets. The number of piglets born effective was calculated by subtracting the IUGR, stillborn, and mummified piglets from the total born. Litter birth weights based on being born alive, but without being stillborn or mummified, were measured immediately after birth, and the individual average birth weight of newborn piglets alive was calculated.

### Statistical Analysis

In the present study, a total of 111 post-weaning sows made up of 56 and 55 sows from the CON and SF groups, respectively, were used to examine their reproductive performances, and 17 sows (10 and seven from the CON and SF groups) from Parity two, 20 sows (9 and 11) from Parity three, 13 sows (6 and 7) from Parity four, and 15 sows (8 and 7) from Parity five were excluded from the analysis of reproductive data. Reproductive traits were assessed for normality and were then analyzed using an unpaired *t*-test (SAS Institute, Cary, NC, USA) using sow as an experimental unit. A chi-square test was used to analyze the percentage of removal for several reasons. Data were presented as means ± SEM. Statistical significance was declared when *p* < 0.05.

## Results

### Culling of Sows

The percentage of sows culled for several reasons is shown in Table 2. There were 111, 94, 74, and 61 sows in the second, third, fourth, and fifth parities, respectively. A total of 33 and 32 sows were culled from Parities two to five for CON and SF, respectively. At each parity, the percentage of the sows culled due to reproductive disorder, low performance, illness, and other reasons were not affected by dietary treatment at the replacement phase between the two groups (*p* > 0.05).

**Table 2:** Number and reason of removal sows at different parities^a, b, c^.

	**CON**	**SF**	***P*** **- value**
**Parity 2**			
Initial number of at post-weaning	56	55	>0.05
Number of pregnant	50	51	>0.05
Number of farrowed	46	48	>0.05
**Numbers and reasons of culling**			
Reproductive disorder	5	1	>0.05
Low performance	3	2	>0.05
Illness	1	3	>0.05
Other reasons	1	1	>0.05
Total	10	7	>0.05
**Parity 3**			
Initial number of at post-weaning	46	48	>0.05
Number of pregnant	39	40	>0.05
Number of farrowed	37	37	>0.05
**Numbers and reasons of culling**			
Reproductive disorder	3	5	>0.05
Low performance	3	1	>0.05
Illness	1	3	>0.05
Other reasons	2	2	>0.05
Total	9	11	>0.05
**Parity 4**			
Initial number of at post-weaning	37	37	>0.05
Number of pregnant	34	33	>0.05
Number of farrowed	31	30	>0.05
**Numbers and reasons of culling**			
Reproductive disorder	2	2	> 0.05
Low performance	2	1	>0.05
Illness	1	2	>0.05
Other reasons	1	1	>0.05
Total	6	7	>0.05
**Parity 5**			
Initial number of at postweaning	31	30	>0.05
Number of pregnant	29	28	>0.05
Number of farrowed	23	23	>0.05
**Numbers and reasons of culling**			
Reproductive disorder	1	1	>0.05
Low performance	2	1	>0.05
Illness	3	4	>0.05
Other reasons	2	1	>0.05
Total	8	7	>0.05

^a^*A chi-square test was used to analyze the data*.

^b^*Reproductive data of the culling sows were excluded from statistical analysis*.

^c^*Con, control diet; SF, control diet supplemented with 0.8% soluble fiber*.

### Plasma Concentrations of AMH

The plasma concentrations of AMH are shown in Table 3. Circulating concentrations of AMH were greater in SF gilts than in CON gilts at 205 d of age at 11.11 ± 0.74, compared with 13.87 ± 1.02 ng/ml (*p* = 0.040) and at the first post-weaning day at 9.02 ± 0.68 compared with 11.26 ± 0.72 ng/ml (*p* = 0.034).

**Table 3:** Effects of soluble fiber (SF) supplementation during the replacement phase on plasma concentration of anti-mullerian hormone (AMH) (ng/ml)^a^.

	**CON**	**SF**	***P*** **value**
On 205 d of age	11.11 ± 0.74	13.87 ± 1.02	0.040
On the day of first post-weaning	9.02 ± 0.68	11.26 ± 0.72	0.034

^a^*Con, control diet; SF, control diet supplemented with 0.8% soluble fiber; AMH, anti-Mullerian hormone; n = 12 per group*.

### Backfat Thickness

The backfat thickness of sows at mating in each parity is shown in Table 4. There was no difference in backfat thickness at mating at Parities two to five between the CON and SF groups (*p* > 0.05).

**Table 4:** Backfat thickness of sows at mating at each parity^a^.

	**CON**	**SF**	***P*** **value**
In parity 2			
No. of sows	32	33	-
BF at mating	15.2 ± 0.43	15.3 ± 0.59	0.838
In parity 3			
No. of sows	27	26	-
BF at mating	14.0 ± 0.47	14.1 ± 0.53	0.814
In parity 4			
No. of sows	24	24	
BF at mating	13.4 ± 0.86	14.2 ± 0.62	0.453
In parity 5			
No. of sows	19	20	-
BF at mating	13.6 ± 0.67	13.7 ± 0.66	0.934

^a^*Con, control diet; SF, control diet supplemented with 0.8% soluble fiber; BF, backfat thickness*.

### Reproductive Data

The litter performance of sows from Parities two to five is presented in Table 5. The number of sows farrowed in CON and SF groups at Parity two was 46 and 48, respectively. The number of piglets for total born, born alive, born effective, stillborn, IUGR, mummy and abnormality, litter weight, or average birth weight alive, was not affected by dietary treatment at the replacement phase (*p* > 0.05).

**Table 5:** Effects of SF supplementation during the replacement phase on reproductive performance from Parities 2 to 5^a, b^.

	**CON**	**SF**	***P*** **value**
**Parity 2**			
No. of sows	46	48	-
No. of total born	10.57 ± 0.42	11.23 ± 0.39	0.244
No. of born alive	9.59 ± 0.46	10.44 ± 0.35	0.146
No. of born effective	9.24 ± 0.44	10.02 ± 0.34	0.164
No. of still born	0.65 ± 0.12	0.50 ± 0.14	0.421
No. of IUGR	0.46 ± 0.10	0.48 ± 0.09	0.873
No. of mummy and abnormality	0.22 ± 0.12	0.29 ± 0.10	0.632
Litter weight, kg	13.35 ± 0.66	14.1 ± 0.43	0.336
Average birthweight, kg	1.42 ± 0.04	1.37 ± 0.03	0.343
**Parity 3**			
No. of sows	37	37	
No. of total born	10.92 ± 0.42	12.24 ± 0.48	0.044
No. of born alive	10.08 ± 0.44	11.51 ± 0.43	0.023
No. of born effective	9.57 ± 0.45	10.97 ± 0.40	0.022
No. of still born	0.62 ± 0.16	0.54 ± 0.15	0.712
No. of IUGR	0.35 ± 0.11	0.54 ± 0.14	0.299
No. of mummy and abnormality	0.27 ± 0.07	0.15 ± 0.07	0.499
Litter weight, kg	15.24 ± 0.71	16.52 ± 0.62	0.180
Average birthweight, kg	1.53 ± 0.05	1.45 ± 0.05	0.274
**Parity 4**			
No. of sows	31	30	
No. of total born	11.50 ± 0.45	12.60 ± 0.34	0.058
No. of born alive	10.75 ± 0.38	12.03 ± 0.37	0.019
No. of born effective	10.44 ± 0.41	11.50 ± 0.33	0.049
No. of still born	0.44 ± 0.13	0.33 ± 0.13	0.410
No. of IUGR	0.31 ± 0.10	0.53 ± 0.16	0.195
No. of mummy and abnormality	0.31 ± 0.14	0.19 ± 0.07	0.592
Litter weight, kg	15.81 ± 0.56	17.45 ± 0.57	0.045
Average birthweight, kg	1.49 ± 0.03	1.47 ± 0.05	0.737
**Parity 5**			
No. of sows	23	23	
No. of total born	11.22 ± 0.47	12.65 ± 0.63	0.075
No. of born alive	10.30 ± 0.48	11.83 ± 0.59	0.067
No. of born effective	10.0 ± 0.43	11.65 ± 0.53	0.020
No. of still born	0.57 ± 0.21	0.61 ± 0.21	0.988
No. of IUGR	0.30 ± 0.13	0.44 ± 0.27	0.321
No. of mummy and abnormality	0.35 ± 0.23	0.32 ± 0.09	0.902
Litter weight, kg	15.32 ± 0.66	16.89 ± 0.72	0.115
Average birthweight, kg	1.51 ± 0.05	1.43 ± 0.05	0.225

^a^*Piglets with a birth weight 69% lower than the average were classified as IUGR*.

^b^*Con, control diet; SF, control diet supplemented with 0.8% soluble fiber*.

Both groups of CON and SF had 37 sows farrowed at Parity three, and the gilts receiving SF diet at the replacement phase had 1.32 piglets of total born (*p* = 0.044), 1.43 piglets born alive (*p* = 0.023), and 1.40 piglets of born effective (*p* = 0.022) more than gilts receiving the CON diet at the replacement phase, while no effects of dietary treatment on the numbers of stillborn, IUGR piglets, mummy, and abnormality were observed (*p* > 0.05).

The CON and SF groups had 31 and 30 sows farrowed at Parity four, and the gilts receiving SF diet at the replacement phase had 1.1 piglets from total born (*p* = 0.058), 1.28 piglets born alive (*p* = 0.019), and 1.06 piglets of born effective (*p* < 0.05), more than gilts receiving the CON diet at the replacement phase, while dietary treatment had no effects on the numbers of the stillborn, IUGR, mummified, and abnormal piglets (*p* > 0.05).

Both the CON and SF groups had 23 sows farrowed at Parity five, and the gilts receiving the SF diet at the replacement phase had 1.43 piglets from total born (*p* = 0.075), 1.53 piglets of born alive (*p* = 0.067), and 1.65 piglets of born effective (*p* = 0.020), more than gilts, which received CON diet, while dietary treatment had no effect on the numbers of the stillborn, IUGR, mummified, and abnormal piglets (*p* > 0.05).

## Discussion

Our previous study demonstrated that SF treatment during the replacement phase resulted in fewer IUGR and a greater number of born effective in their first parity (9). This study extended the research to investigate the long-term effects of SF supplementation to replacement gilts on adult sow performance. The most important finding was that dietary supplementation of SF during the replacement phase had a long-term benefit on the reproductive performance of breeding sows. The CON sows produced 1,516 total born and 1,395 of born alive from Parities two to five and the SF treatment during the replacement phase resulted in 1,661 total born and 1,560 born alive, and the lifetime reproductive performance and culling rate could be predicted by the number of the piglets born alive at Parity one in both high- and low-performing herds in eastern Asia (6, 7). An early litter size trait could be considered as an indirect selection trait for longevity and to estimate genetic parameters (19). The increase in age at first mating might decrease the longevity and lifetime reproductive efficiency of sows in breeding herds (20). The current findings, combined with the existing evidence, suggested that successful rearing of replacement gilts was important for their lifetime performance.

The long-term benefits of the SF supplementation during the replacement phase could be attributed to several reasons. The dietary fiber treatment during the replacement phase resulted in numerous ovarian follicles (17, 18) and enhanced oocyte quality (21, 22). When gilts were fed with graded levels of inulin and cellulose from 92 d of age, the number of healthy ovarian follicles was linearly increased and the number of total follicle reserve per ovary increased by 40% when gilts were fed 100% more dietary fiber than the control gilts (17), and the number of atretic follicles was linearly decreased as the increase of the dietary fiber level (17). Dietary fiber supplementation to replacement gilts from the age of 161 d could protect against high-energy feeding-induced ovarian follicle loss (18). The AMH is secreted from the ovarian granulosa cells in the growing follicles, which, in turn, inhibits the cyclic recruitment of antral follicles by influencing the follicle-stimulating hormone (FSH) threshold levels and, therefore, plays a follicle-preserving function for pigs (23). Given the assumed role of AMH in predicting the size of the ovarian follicle pool and AMH as a marker of future reproductive success (16), the serum level of AMH was measured in non-pubertal gilts at the age of 205 d, and it was found that the SF gilts had a significantly higher level of AMH than the control gilts. The level of AMH was also measured in the gilts at their first post-weaning, and it was found that the gilts with SF consumption at the replacement phase had greater levels of AMH than that in the CON group. In humans, AMH concentrations were observed to be positively associated with intake of dietary fiber (24). The plasma AMH levels at the prepubescent gilts were found to be greater than in post-weaning sows, attributed to the inhibiting influence of progesterone and prolactin on the activation of primordial and developing follicles (25). The greater level of AMH in the gilts or sows of the SF group indicated that the enhanced ovarian follicular function might predict a larger reproductive performance in the subsequent cycles (16), and the long-term fertility could be affected by the immune system (26, 27). It was observed that dietary fiber improved the litter size of sows, which were associated with alternation of immune function (28). The supplementation of spray-dried porcine plasma, an ingredient known for its immune modulation function, has long-term merit for reducing stillborn pigs and benefits litter size in the next parities (29), and the nutrient dietary fiber has been shown to exert its benefits *via* immunity (29–32). However, this hypothesis needs further investigation.

The SF treatment to replacement gilts did not influence the removal of sows at different parities. In the present study, 111 gilts with 56 in the CON group and 55 in the SF group became pregnant at Parity one and entered the reproductive cycle and 46 of them became pregnant at Parity five, with 23 remaining in each group, with the overall removal of 59% representative of rates observed in southern China (3, 11). However, the dietary treatment during the replacement phase did not affect the removal of sows at different parities, possibly because the reasons for the culling of the sows were complex as shown in Table 2, and the number of sows was not large enough to achieve a solid conclusion.

This study had some limitations. The reproductive performance of the sows was not only represented by the litter performance but also the suckling performance, including pre-weaning survival and weaning weight, which was not shown in the present study, and different litter sizes would result in differences in body mobilization (33) and BW at post-weaning, which, in turn, affects the subsequent performance (34). The body weight changes were not detected along the experimental period, but the backfat thickness was measured as an alternative, and it was found that dietary treatment did not affect the backfat thickness. Lastly, the diets fed to the gestating and lactation sows from Parities two to five were the same as that in Parity one, and intakes of some nutrients were larger than the comments of NRC standards 2012 (35).

## Conclusion

Collectively, the present results demonstrated that SF supplementation during the replacement phase benefited litter size in the subsequent parities and confirmed that the nutritional decisions replacement phase could exert a long-term effect in the subsequent parities as breeding sows.

## Data Availability Statement

The raw data supporting the conclusions of this article will be made available by the authors, without undue reservation.

## Ethics Statement

The animal study was reviewed and approved by all aspects of the experimental protocol of this study were in accordance with the animal care and use Committee of Sichuan Agricultural University.

## Author Contributions

DW and YZ designed and supervised the experiments and wrote the manuscript. YZ and LH conducted the animal trial and performed data collection. SX and JL conducted statistical analyses. LC, YL, BF, and DW supervised this trial. All authors contributed to the article and approved the submitted version.

## Funding

This work was supported by the National Natural Science Foundation (31772616) of the People's Republic of China.

## Conflict of Interest

The authors declare that the research was conducted in the absence of any commercial or financial relationships that could be construed as a potential conflict of interest.

## Publisher's Note

All claims expressed in this article are solely those of the authors and do not necessarily represent those of their affiliated organizations, or those of the publisher, the editors and the reviewers. Any product that may be evaluated in this article, or claim that may be made by its manufacturer, is not guaranteed or endorsed by the publisher.
